# Development of a duplex chamber digital PCR to quantify twelve genetically modified maize events

**DOI:** 10.1080/21645698.2025.2548053

**Published:** 2025-08-21

**Authors:** Eun-Ha Kim, Youn-Sung Cho, Byeori Kim, Yelinn Yoo, Jae-In Lee, Young Soon Kim, Tae-Sung Park

**Affiliations:** a Biosafety Division, National Institute of Agricultural Sciences, Jeonju, Korea; b Planning & Coordination Division, National Institute of Agricultural Sciences, Jeonju, Korea

**Keywords:** Chamber digital PCR, genetically modified maize, quantitative method

## Abstract

Accurate quantification of genetically modified organisms (GMOs) is essential for regulatory compliance, especially under threshold-based labeling policies. In this study, we developed and validated twelve event-specific duplex chamber- or chip-based digital PCR (cdPCR) methods using microfluidic array plates to quantify GM maize events approved in South Korea. In contrast to conventional real-time PCR, the cdPCR approach allows for absolute quantification without standard curve calibration and incorporates event-specific zygosity ratio correction to improve accuracy of the measurement. The method was evaluated at GMO content levels of 0.9%, 3.0%, and 5.0%. It demonstrated high sensitivity and robustness, with trueness, precision, and reproducibility satisfying the minimum performance criteria recommended by international guidelines. Comparative analysis with real-time quantitative PCR (qPCR) showed comparable accuracy; however, cdPCR provided advantages in cost-efficiency and operational simplicity. These findings support the applicability of duplex cdPCR as a practical and reliable tool for GMO quantification, particularly in national regulatory laboratories and for enforcement of labeling thresholds such as Korea’s 3.0% rule.

## Introduction

The advancement of genetically modified (GM) crops has been significantly driven by scientific and technological developments, resulting in traits such as pest resistance, herbicide tolerance, resilience to environmental stress, and enhanced nutritional profiles.^1,2^ In 2024, GM crops were cultivated on approximately 209.8 million hectares globally. Among these, GM soybeans accounted for 50.0% of the total cultivation area, followed by GM maize (32.5%), GM cotton (11.8%), and GM canola (5.0%).^3^

To ensure consumer choice, transparency, and harmonized trade practices, many countries have implemented labeling regulations that require quantification of GM content based on predefined thresholds. For example, South Korea enforces a GMO labeling threshold of 3.0% for food,^4^ while the European Union and Japan apply thresholds of 0.9% and 5.0%, respectively.^5–7^

Accurate and reliable quantification methods are essential for verifying compliance with such labeling requirements. GM content in food or feed is generally expressed as the ratio of transgene (GM-specific target) to an endogenous reference gene. Currently, real-time quantitative PCR (qPCR) is the most widely used technique for GMO quantification.^8–12^ However, qPCR has inherent limitations, including its dependence on standard curves and vulnerability to amplification efficiency variations and PCR inhibitors.^13–15^

As an alternative, digital PCR (dPCR) has emerged as a next-generation method for absolute quantification of nucleic acids. In dPCR, the reaction mixture is partitioned into thousands of independent reactions, and after amplification, each partition is classified as positive or negative. The absolute quantity of the target is then calculated using Poisson statistics.^16,17^ One major advantage of dPCR over qPCR is that it enables absolute quantification without the need for standard curve calibration, thereby improving reproducibility and reducing operator dependency, particularly when working with low target concentrations.^18,19^ Due to these advantages, dPCR has been widely applied to the detection and quantification of GMOs in recent years.

dPCR platforms are broadly classified into droplet-based dPCR (ddPCR) and chamber- or chip-based dPCR (cdPCR) systems.^16,19,20^ ddPCR offers high partitioning resolution and enhanced sensitivity for detecting low-copy-number targets. It also enables flexible multiplexing through amplitude-based fluorescence clustering.^15,21,22^ By contrast, cdPCR platforms simplify operation by eliminating droplet generation and emulsification steps. Their pre-defined microchamber structure ensures uniform partition volumes, improves thermal uniformity, and minimizes cross-contamination risks. Notably, cdPCR systems support image-based fluorescence detection, allowing precise duplex or multiplex quantification with reduced signal overlap.^16^

Employing these platform-specific advantages, various dPCR assays have been developed for the simultaneous quantification of multiple transgenic elements, demonstrating high accuracy, sensitivity, and wide dynamic range.^15,23–25^ In support of this progress, the European Network of GMO Laboratories and other expert groups have established performance criteria and validation guidelines specifically tailored for dPCR-based GMO quantification.^19,20,26^

In South Korea, the safety assessment and management of GMOs are regulated under the Act on Transboundary Movements of Living Modified Organisms.^27^ The Rural Development Administration is responsible for conducting environmental risk assessments for agricultural GMOs. To support this regulatory framework, the Biosafety Division of the National Institute of Agricultural Sciences (NIAS) verifies detection and quantification methods using qPCR and dPCR techniques for GM crops approved domestically. These validations follow internationally recognized protocols, including those provided by the European Union Reference Laboratory for GM Food and Feed (EURL-GMFF).^9,12,26,28–30^

The objective of this study is to develop and validate a duplex cd PCR method for the absolute quantification of twelve GM maize events approved in South Korea. The selection of the 12 GM maize events in this study was based on their regulatory approval for food, feed, or industrial use in South Korea and their relevance to ongoing domestic monitoring programs. The assay aims to support regulatory enforcement of the 3.0% labeling threshold for GMOs^4^ by enabling precise quantification without calibration curves and by incorporating experimentally determined zygosity ratios for each GM event. We applied the QuantStudio Absolute Q™ system, which simplifies the dPCR workflow by using pre-manufactured microfluidic chamber plates. Furthermore, our method was evaluated through blind sample testing at regulatory threshold levels (0.9%, 3.0%, and 5.0%) using both cdPCR and qPCR. The results demonstrate that duplex cdPCR is a robust and practical approach for accurate GMO quantification in support of national monitoring and inspection efforts.

## Materials and Methods

### Plant Materials

Twelve GM maize events – DAS01131-3, DAS40278-9, DP004114-3, DP023211-2, DP051291-1, DP098140-6, DP202216-6, MON87403-1, MON87411-9, MON87429-9, MON87460-4, and MON88017-3—and their corresponding non-GM reference materials were provided by the respective developers for in-house method verification conducted at the Biosafety Division of the NIAS, South Korea. Detailed information on each GM maize event, including the approval year in South Korea, intended use (food, feed, or industrial), trait characteristics, and developer, is provided in Supplemental Table S1.

### Genomic DNA Extraction

Maize seeds (both GM and non-GM) were ground into a fine powder using a mortar and pestle under liquid nitrogen. Genomic DNA was extracted and purified using the Maxwell RSC PureFood GMO and Authentication Kit (Promega, Madison, WI, USA), following the manufacturer’s instructions. DNA concentration and purity were assessed using a NanoDrop One spectrophotometer (Thermo Fisher Scientific, Waltham, MA, USA), and double-stranded DNA content was quantified using a Qubit 4 Fluorometer (Thermo Fisher Scientific).

### Primers and Probes

Event-specific primers and probes, as well as those targeting the endogenous reference gene *high mobility group a* (*hmg*, GenBank accession no. AJ131373.1), were provided by the respective applicants in accordance with the national regulatory requirements under the LMO ACT of South Korea, which obliges GM developers to submit molecular characterization and detection information for official method verification. The Sequences and target regions are listed in Table 1. They are also publicly available in international databases such as International Service for the Acquisition of Agri-biotech Applications (ISAAA) and EUginus. For certain GM maize events such as DP004114–3 and DP023211–2, the primers and probes used in this study differ from those specified in the EURL-GMFF validated methods (e.g., QT-EVE-ZM-035 and QT-TAX-ZM-008). Probes targeting GM events were labeled with 6-carboxyfluorescein (FAM), while *hmg* probes were labeled with either FAM or VIC. The 3′ ends of the probes were modified with either TAMRA or non-fluorescent quencher-minor groove bindeer (NFQ-MGB). All oligonucleotides were synthesized by Bioneer (Daejeon, South Korea).

**Table 1.** Primers and probes used for GM maize event-specific targets and the endogenous reference gene (*hmg*).

Event name	Primers/probes sequence (5’-3’)		^a^Length (bp)
DAS01131–3		F- CTA AGA GCT AAG ATT GCG CGG *R*- TTC GGG CCT AAC TTT TGG TG *p*- [6-FAM]-ACA TAT TTT TTG AGG ATA ACA GCA-[NFQ-MGB]	98
DAS40278–9		F- CAC GAA CCA TTG AGT TAC AAT C *R*- TGG TTC ATT GTA TTC TGG CTT TG *p*- [6-FAM]- AGC TAA CCT TCA TTG TAT TCC G-[TAMRA]	98
DAS59122–7		F- GGG ATA AGC AAG TAA AAG CGC TC *R*- CCT TAA TTC TCC GCT CAT GAT CAG *p*- [6-FAM]- TTT AAA CTG AAG GCG GGA AAC GAC AA-[TAMRA]	86
DP 004114–3		F- CGT TTG TAG CAC TTG CAC GTA GT *R*- GGT AAC CGC TCT TCC AGT TGA A *p*- [6-FAM]- AAG CTT CAA CAC AGA TC-[NFQ-MGB]	90
DP023211–2		F- CAT TTT GGA TTG TAA TAT GTG TAC CTC A *R*- CTA GCT CGA CTA GTT AGT TAG ACG CG *p*- [6-FAM]- TTA AAT CTG ACG TGA GGC GC-[NFA-MGB]	92
DP098140–6		F- GTG TGT ATG TCT CTT TGC TTG GTC TT *R*- GAT TGT CGT TTC CCG CCT TC *p*- [6-FAM]- CTC TAT CGA TCC CCC TCT TTG ATA GTT TAA ACT-[TAMRA]	80
DP202216–6		F- CCA TCT GAG GTC TGC ACT CTC AC *R*- CTC CGC TCA TGA TCA GAT TGT C *p*- [6-FAM]- CAA CAC ACT CAA ACA CTG ATA G-[NFQ-MGB]	105
MON87403–1		F- CTT TCT TTT TCT CCA TAT TGA CCA TCA TAC *R*- TAC TCC GGA ATG AGT GCT CTG TAT C *p*- [6-FAM]- TCA TTG CGA TCC ACA TTT CCC TAC ATG G-[TAMRA]	88
MON87411–9		F- CTC TGT AAC AGA AAA CAC CAT CTA GAG *R*- ACA AAA GTG AAC TAG TTC TAG GGT AGA T *p*- [6-FAM]- CCG CGT TTA AAC TAT CAG TGT TTA GAG AAT-[TAMRA]	109
MON87429–9		F- CGA GAC AGA CTC AAT GTA TCC GAG ATA CTC *R*- CCA TCA TAC TCA TTG CTG ATC CAT GTA *p*- [6-FAM]- TCC CGG ACA TGA AAC CAA ACA AGA GTG GTC-[TAMRA]	116
MON87460–4		F- CAC GTT GAA GGA AAA TGG ATT G *R*- TCG CGA TCC TCC TCA AAG AC *p*- [6-FAM]- AGG GAG TAT GTA GAT AAA TTT TCA AAG CGT TAG ACG GC-[TAMRA]	82
MON88017–3		F- GAG CAG GAC CTG CAG AAG CT *R*- TCC GGA GTT GAC CAT CCA *p*- [6-FAM]- TCC CGC CTT CAG TTT AAA CAG AGT CGG GT-[TAMRA]	94
*hmg* (endogenous reference gene)		F- TTG GAC TAG AAA TCT CGT GCT GA *R*- GCT ACA TAG GGA GCC TTG TCC T *p*- [VIC]-CAA TCC ACA CAA ACG CAC GCG TA-[NFQ-MGB]	79

^a^Length denotes the amplicon size in base pairs. In primer/probe sequence, F, R, and P indicate forward primer, reverse primer, and probe, respectively. TAMRA, 6-carboxytetramethylrhodamin NFQ-MGB, non fluorescent quencher-minor groove binder.

### Real-Time qPCR Analysis for DP023211–2

Quantitative PCR (qPCR) was conducted using the QuantStudio 1 Real-Time PCR Instrument (Applied Biosystems by Thermo Fisher, Foster City, CA, USA) to establish calibration curves for the DP023211–2 event. Each 25 μL reaction contained 12.5 μL of 2X Qplex Master Mix (CellSafe, Yougin, South Korea), primers, probes, 10 μL of template DNA, and nuclease-free water. The reagents are detailed in Supplemental Table S2. Two zygosity ratios were applied to calibrate the DNA mixtures: 0.42 (provided by the applicant) and 0.13 (experimentally determined by cdPCR in this study), to simulate 10% GM content based on copy number ratios. The calibration curves were established on at five samples with four experimental replicates. The first point of the calibration curve (S1) was established for a sample containing 10% maize DP023211–2 DNA in a total of 300 ng of non-GM maize DNA. Standards S2 to S5 are prepared by serial dilutions (dilution factor 4 for S2, S4 and S5, and dilution factor 3 for S3). The corresponding amount of DNA in reaction and copy numbers for standard curves are listed in Supplemental Table S3.^31^ Thermal cycling conditions were 95°C for 10 min, followed by 45 cycles of 95°C for 15 s and 60°C for 1 min. The same thermal protocol was used for both the DP023211–2 and *hmg* assays. Blind samples containing 0.9%, 3.0%, and 5.0% GM content were prepared by mixing DP023211–2 DNA with non-GM maize DNA at the corresponding copy number ratios. Copy numbers were calculated from Ct values based on the standard curves. GMO content was expressed as the ratio of DP023211–2 to *hmg* copy numbers. Accuracy was evaluated as relative bias, and precision was assessed as the relative standard deviation (RSD) from four technical replicates.

### cdPCR Analysis

Simplex and duplex cdPCR assays were performed using the QuantStudio Absolute Q™ Digital PCR System (Applied Biosystems by Thermo Fisher) with 16-well microfluidic array plates (MAP). Reaction mixtures followed the manufacturer’s protocol. For simplex assays, each 9 μL reaction included 2 μL of 5X Absolute Q™ DNA Digital PCR Master Mix, 5 μL of template DNA (1 ng/µl), 0.9 μL of primer mix (10 μM), 0.25 μL of probe (10 μM), and 1.4 μL of distilled water. For duplex assays, the same master mix and DNA volumes were used, with two sets of primers and probes (total 2.3 μL), and 0.7 μL of water. Each well was loaded with 9 μL of reaction mixture and overlaid with 15 μL of separation oil (Thermo Fisher Scientific, A52730), followed by placement of a sealing gasket. Each well contained 20,480 fixed microchambers. Thermal cycling was carried out at 96°C for 5 min, followed by 40 cycles of 96°C for 5 s and 60°C for 15 s. Fluorescence signals were analyzed using the QuantStudio 3D Digital PCR instrument and QuantStudio Analysis Suite Cloud Software. Zygosity ratios for each GM event were calculated using duplex cdPCR by measuring the ratio of event-specific target copy number to *hmg* copy number in GM DNA samples. Blind DNA mixtures at 0.9%, 3.0%, and 5.0% GM content were analyzed to evaluate the method’s performance in quantifying low-level GMO content using cdPCR. Four experimental replications were run for each cdPCR analysis.

## Results and Discussion

### Comparison of Simplex and Duplex cdPCR Analysis

GMO quantification is based on the ratio of transgene to endogenous gene copy numbers. Therefore, simultaneous detection within a single reaction mixture using duplex PCR is both practical and cost-effective. To evaluate this, we compared the performance of simplex and duplex cdPCR for two GM maize events: DAS01131–3 and DP910521–2. In the simplex assay, the FAM reporter dye was used to detect both the transgene and the *hmg* endogenous gene. The measured copy numbers for DAS01131–3 and DP910521–2 transgenes were 439.09 and 427.45 copies/μL, respectively, while *hmg* copy numbers were 1048.41 and 1174.39 copies/μL (Table 2). To assess any dye-related bias, *hmg* quantification was performed using both FAM and VIC dyes. The results showed no statistically significant difference between the two fluorophores (FAM, 1048.41 copies/μL; VIC, 1077.4 copies/μL), as determined by a Student’s t-test (*p* > .05), indicating that dye selection did not influence *hmg* quantification.

**Table 2.** Quantitative results of simplex and duplex cdPCR assays for DAS01131–3 and DP051291–2 event^a^.

Event	System	Target	Dye	Total	Positive	Conc. Cp/µl	SD
DAS01131–3	simplex	GM	FAM	20408	3526	439.09	20.88
	simplex	*hmg*	FAM	20451.8	7448	1048.41	37.59
	simplex	*hmg*	VIC	20432.5	7604	1077.4	19.0
	duplex	GM	FAM	20424.5	3453	428.74	6.86
		*hmg*	VIC		7455	1051.3	20.50
DP051291–2	simplex	GM	FAM	20428.8	3443	427.45	18.68
	simplex	*hmg*	FAM	20407.3	8135	1174.39	18.82
	simplex	*hmg*	VIC	20437.3	8152	1177.98	24.56
	duplex	GM	FAM	20421.3	3329	411.74	21.25
		*hmg*	VIC		8038	1157.12	22.24

^a^Each value represents the average of four experimental replications, except for SD. SD, standard deviation of Conc. Cp/µl (concentration of copy/µl).

Accurate digital PCR requires efficient partitioning. The MAP 16 Digital PCR Plate used in this study contains 20,480 fixed microchambers per well. The observed positive chamber counts exceeded 20,400, indicating even partitioning. In duplex cdPCR, FAM was used for transgene detection and VIC for *hmg*. The resulting copy numbers were statistically comparable to those obtained from simplex assays, with no significant difference observed based on Student’s t-test (*p* > .05), confirming that amplification efficiency was not influenced by dye type or reaction format. These findings validate the use of duplex cdPCR for GMO quantification.

### Determination of Zygosity Ratio in GM Maize Events Using cdPCR

Determination of the zygosity ratio of the non-GM maize is critical for expressing GM content as a haploid genome copy ratio, as emphasized in EU guide lines.^32–34^ Maize has a complex tissue structure with variable DNA contributions from maternal and paternal genomes, depending on the hybrid type. The zygosity ratio can vary depending on the donor parent of the transgene and specific seed tissue composition, such as the embryo, endosperm, and seed coat.^35^ Corbisier et al.^34^ reported that the copy number ratios between GM event-specific targets and taxon-specific reference genes varied notably among 17 certified GM maize reference materials using ddPCR. The observed conversion factors ranged from 0.34 to 0.68, depending on the parental origin of the transgene (male vs. female donor), endosperm contribution, and potential differences in DNA extractability from seed tissues. In line with these findings, our study also showed the zygosity ratio ranged from 0.134 (DP023211–3) to 0.625 (DP202216–6) (Table 3), indicating hemizygous GM maize materials.

**Table 3.** Zygosity ratio of GM event-specific and endogenous *hmg* targets in positive control samples as determined by cdPCR^a^.

Event	Total	Event specific			Endogenous Reference			Zygosity ratio
		Positive	Conc.cp/µl.	SD	Positive	Conc.cp/µl.	SD	
DAS01131–3	20424.75	3402	421.77	10.38	7495	1058.35	11.69	0.399
DAS40278–9	20441	5227	683.55	4.34	10521	1674.37	59.25	0.408
DAS59122–7	20198.75	4606	598.82	42.58	9232	1413.77	80.47	0.424
DP004114–3	20413	5524	732.52	104.5	12023	2058.52	43.68	0.356
DP023211–2	20406	2024	241.77	5.23	11039	1802.55	40.66	0.134
DP098140–6	20431.5	6824	941.1	41.67	10724	1722.73	16.81	0.546
DP202216–6	20446.75	8510	1245.86	24.78	11803	1993.23	40.51	0.625
MON87403–1	20457.75	3984	501.36	12.23	6347	859.81	13.99	0.583
MON87411–9	20395.75	3953	498.64	1.75	6167	833.55	10.58	0.598
MON87429–9	20423.25	3301	408.26	35.19	5942	795.8	9	0.513
MON87460–4	20409.75	3129	385.24	8.23	6593	903.02	14.6	0.427
MON88017–3	20415	3247	400.99	8.91	5775	769.75	10.2	0.521

^a^Each value represents the average of four experimental replications, except for SD. SD, standard deviation of Conc. Cp/µl (concentration of copy/µl).

Our results were compared with zygosity ratio values available in the applicant’s reference protocols and the validation report of the GM maize event by the EURL-GMFF (Supplemental Table S4). Among the 12 GM maize events tested, the most notable discrepancies between experimentally determined and reference zygosity ratios were observed in DP023211–2 and DP202216–6. For DP023211–2, the duplex cdPCR method yielded a zygosity ratio of 0.134 (mean of four replicates), while the applicant’s documentation reported a value of 0.42. Similarly, DP202216–6 showed experimentally determined ratios of 0.625, compared to values of 0.99 reported by the EURL-GMFF. In these cases, the reference values were provided as single figures without replicate data or accompanying measures of variability (e.g., standard deviation), precluding formal statistical comparison (e.g., t-test). Nevertheless, the relative differences – ranging from 36% to over 68%—are substantial and analytically meaningful. These discrepancies likely reflect differences in seed batch composition, parental genotype, hybrid type, or tissue-specific DNA content, all of which can influence the effective zygosity ratio observed in quantitative PCR-based analyses.^34^ To account for such variation, this study applied duplex cdPCR to determine zygosity ratios directly from seed-derived DNA, enabling a material-specific correction for each event. This approach ensures more accurate quantification when CRMs are unavailable, not matrix-matched, or exhibit batch-to-batch variability.

### Quantification of DP02311–3 Blind Samples by qPCR

**Figure 1.**
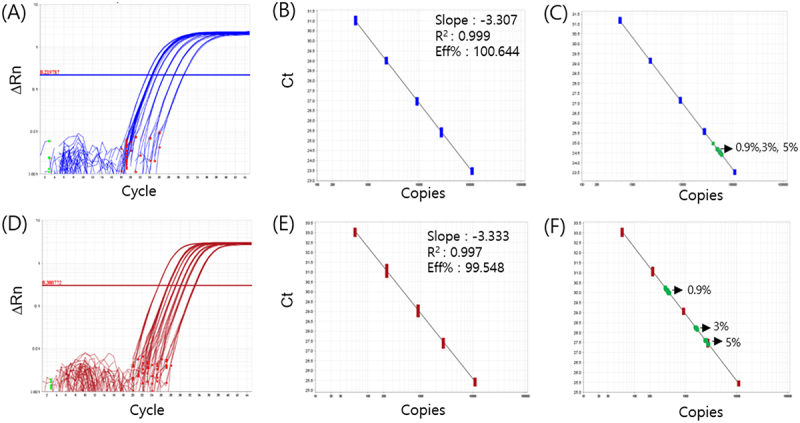
Detection of DP023211–2 blind samples using qPCR analysis. (A) Amplification plots for calibrators and blind samples. (B) Standard curves for *hmg* with concentrations ranging from 572 to 109,890 copies. (C) DP023211–2 blind samples with expected transgene contents of 0.9%, 3.0%, and 5.0% on the *hmg* standard curve (D) standard curves for DP023211–2 with concentrations ranging from 57 to 10,989 copies. (E) DP023211–2 blind samples expected transgene contents of 0.9%, 3.0%, and 5.0% on the DP023211–2 standard curve. A zygosity ratio of 0.13 was applied to calculate the copy number ratio. Data were means from four replicates.

To further investigate the impact of the zygosity ratio on quantification, we established a standard curve using qPCR with DNA templates adjusted to match the expected copy ratios corresponding to the two distinct zygosity ratios of DP023211–2. The average slope of the standard curve should range from −3.1 to −3.6, corresponding to an efficiency between 90% and 110%, and the R^2^ coefficient should be ≥ 0.99, according to the European Network of GMO Laboratories (ENGL) method acceptance criteria (2008). When using the zygosity ratio of 0.43 provided by the applicants, the slope, R^2^, and efficiency values met the ENGL criteria. However, the Ct value showed high variation at the lowest concentration (S5) of DP023211–2 (Supplemental Fig. S1D). In contrast, when using the zygosity ratio of 0.13 measured via cdPCR in this study, the slope, R^2^, and efficiency values met the ENGL criteria, and the Ct value exhibited less variation across the five concentrations (Figure 1(B,E)). These results indicate that a zygosity ratio of 0.43 led to an underestimation of GM quantity, affecting PCR amplification efficiency, particularly at the lowest concentration.

The DP02311–3 event-specific and *hmg* qPCR analyses were performed to quantify blind DNA samples with GM levels of 5.0%, 3.0%, and 0.9% (expressed as the % copy number ratio of DP02311–3 to *hmg*). The measured copy numbers of the DP02311–3 event-specific target and *hmg*, as well as the % copy number ratio of DP02311–3 to *hmg*, are shown in Table 4. The measured GMO % was determined as the ratio of the average copy number of DP02311–3 to that of *hmg*.

**Table 4.** Comparison of quantitative performance between qPCR and cdPCR for GM content analysis of DP023211–2 maize event^a^.

	qPCR					cdPCR				
	Copy numbers		Content, %			Copy numbers/µl		Content, %		
DP023211–2	GM	*hmg*	Value	Bias	RSD	GM	*hmg*	Value	Bias	RSD
0.9%	456.3	52691.7	0.87	3.33	1.61	2.3	1848.7	0.93	3.06	17.0
3.0%	1672.9	55540.2	3.01	0.33	2.27	6.7	1726.2	2.90	3.25	17.6
5.0%	2817.5	55507.3	5.09	1.80	4.63	11.1	1736.2	4.78	4.50	6.8

^a^Each value is an average of four experimental replications, except for Bias and RSD. Bias, deviation from expected value; RSD, relative standard deviation.

According to the minimum performance requirements for GMO analytical methods, the measured value must have a relative bias of ± 25% (trueness) and precision in terms of a relative standard deviation (RSD) of ≤ 25%.^26,28^ The measured % copy number ratios were 0.87%, 3.01%, and 5.09%, which were close to the expected values (Table 4). For all blind samples, the % bias and % RSD of the measured copy number ratios were below 5%. The measured copy numbers and Ct values of the DP02311–3 event-specific target in blind DNA samples were well-aligned with the standard curves of *hmg* and DP02311–3 (Figure 1(C,E)), indicating that the qPCR assays precisely measured the % copy number ratio in blind samples.

### Quantification of DP02311–3 Blind Samples by cdPCR

Unlike qPCR, digital PCR (dPCR) enables absolute quantification of the target without the need for a standard curve. Several studies demonstrated that ddPCR platforms provide high analytical performance for regulatory testing of GM events in maize, soybean, and canola.^15,22,36–39^ For instance, Dobnik et al.^21^ developed two multiplex ddPCR assays to quantify up to twelve GM maize events simultaneously, achieving limit of quantification (LOQ) as low as 0.058% and bias within ± 25%. Li et al.^38^ developed an event-specific duplex ddPCR in DBN9936 maize, demonstrating accurate quantification across blind matrix and genomic DNA samples with RSD and bias within ± 25%. While the ddPCR method typically require emulsification steps and specialized droplet readers, in this study the cdPCR method utilizes pre-fabricated microfluidic plates and eliminates droplet generation, thereby reducing operational complexity.

**Figure 2.**
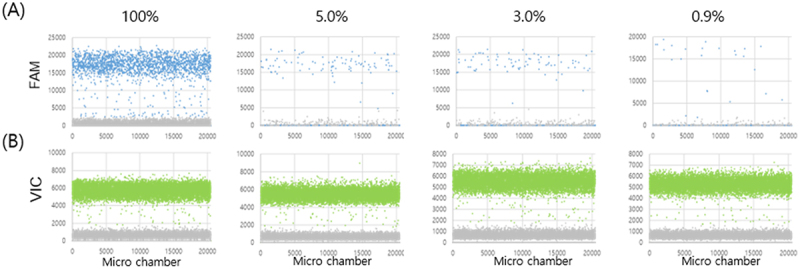
Quantification of the DP023211–2 blind samples using cdPCR analysis. DNA samples from DP023211–2 positive and negative control were mixed to obtain final transgene contents of 100%, 5.0%, 3.0%, and 0.09%. The mixed DNA samples were used as a template to amplify the transgene (A) and *hmg* (B) on the cdPCR platform.

In this study, we established a duplex cdPCR method for quantifying DP02311–3 blind samples. Figure 2 shows the amplification results for DP02311–3 (Figure 2(A)) and *hmg* (Figure 2(B)) at 100%, 5.0%, 3.0%, and 0.9% GMO content. The measured GM % was determined as the ratio of the average copy number of DP02311–3 to that of *hmg*, divided by the zygosity ratio (Table 4). The measured % copy number ratios were 0.93%, 2.90%, and 4.78%, with a % bias of less than 5.0%. The % RSD of the measured copy number ratios for 0.9%, 3.0%, and 5.0% was 17.0%, 17.6%, and 6.8%, respectively, which fell within the acceptable range of ≤ 25%.^28^

**Figure 3.**
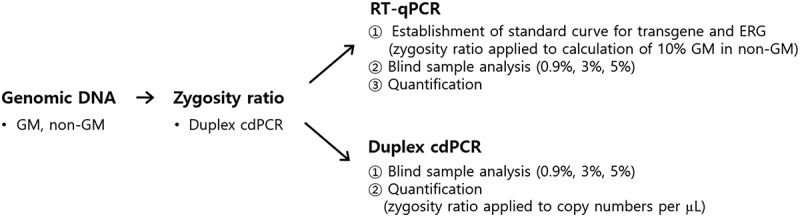
Schematic representation of RT-qPCR and duplex cdPCR analyses for GM maize quantification. ERG, endogenous reference gene.

Both DP02311–3 and *hmg* qPCR and duplex cdPCR accurately quantified GMO content (Table 4). However, the results of this study differ from previous reports suggesting that dPCR exhibits higher precision than qPCR in GMO quantification.^24,38^ In this study, qPCR quantification was conducted after multiple independent runs to adjust DNA concentration, as well as primer and probe concentrations and ratios, to meet the acceptable criteria set by the ENGL.^28^ In contrast, cdPCR analysis was performed without such optimization trials, which may explain the higher precision observed in the qPCR results. Additionally, the amount of template DNA used for the blind samples (5.0%, 3.0%, and 0.9%) in qPCR was 150 ng, 90 ng, and 27 ng, respectively, which was significantly higher than the 0.225 ng, 0.135 ng, and 0.0405 ng used in cdPCR. Therefore, duplex cdPCR is a more cost- and time-efficient method without the need for standard curves or extensive optimization steps. Moreover, it enables accurate quantification even in samples with low GMO content, demonstrating its practical applicability. Schematic representations of the developed qPCR and duplex cdPCR analyses to quantify GM maize content were presented in Figure 3.

### Quantification of Eleven GM Maize Blind Samples by cdPCR

The established cdPCR method was further applied to quantify the GM maize blind samples (0.9%, 3.0%, and 5.0%) in eleven maize events. The results for the target genes (DAS01131-3, DAS40278-9, DP004114-3, DP051291-1, DP098140-6, DP202216–6, MON87403-1, MON87411-9, MON87429-9, MON87460–4, and MON88017-3) and *hmg* cdPCR quantification are shown in Table 5. The GM content was determined as the ratio of the average copy number of the transgene to that of *hmg*, divided by the zygosity ratio (Table 2). The measured GM % for each maize event was similar to the expected value. The precision and trueness of quantification were within the limits established by the ENGL,^28^ except for DAS01131–3 and MON88017–3. The 0.9% DAS01131–3 sample was measured as 0.721%, with an RSD of 27.89% and a bias of −19.88%, while the 0.9% MON88017–3 sample was measured as 0.669%, with an RSD of 30.38% and a bias of −16.41%. Increased % RSD and % bias in PCR-based GMO quantification analyses were observed in samples with concentrations approaching the minimum value tested, which is consistent with previous reports.^8,15,38^ Therefore, further investigation is needed to determine whether increasing the template amount for the 5.0%, 3.0%, and 0.9% blind samples in cdPCR reduces % RSD and % bias. Nevertheless, this study showed that cdPCR achieved accurate and precise quantification of GM content at 3.0%, a GMO labeling threshold in South Korea^4^ without optimization trials, while qPCR required multiple optimization steps (e.g. DNA input, primer/probe concentration) to meet ENGL criteria (data not shown).

**Table 5.** Precision and trueness of GM quantification, corrected by zygosity ratio as determined by cdPCR^a^.

Event		Total	Event specific target			*hmg*					
	^b^Target GM-levels %		Positive	Conc.cp/µl.	SD	Positive	Conc.cp/µl.	SD	^b^Measured GM-level %	Precision (RSD%)	Trueness (bias %)
DAS01131–3	0.9	20426.3	26	2.98	0.57	7373	1037.1	47.96	0.721	27.89	−19.88
	3.0	20455.3	111	12.54	0.61	7563	1068.7	24.23	2.944	6.19	−1.85
	5.0	20445.3	191	21.75	1.49	7742	1101.8	23.17	4.954	9.51	−0.93
DAS40278–9	0.9	20463.3	59	6.71	0.86	10382	1638.8	19.29	1.003	15.69	11.44
	3.0	20474.8	190	21.58	1.22	10296	1618.0	31.34	3.067	3.15	8.90
	5.0	20453.5	294	33.54	0.78	10237	1606.9	27.63	5.113	3.84	2.25
DAS59122–7	0.9	20361	46	6.46	0.61	9208	1393.4	27.13	0.878	11.60	−2.48
	3.0	20437.5	157	27.16	1.5	9147	1373.7	26.96	3.063	3.93	2.09
	5.0	20417.8	288	44.64	2.53	9165	1379.2	18.29	5.620	2.87	12.40
DP004114–3	0.9	20457	48	5.44	0.36	12447	2170.9	47.2	0.704	6.59	−21.76
	3.0	20450.5	176	19.98	1.08	12423	2164.9	41.5	2.593	6.07	−13.55
	5.0	20462.5	285	32.44	2.46	12468	2176.2	54.4	4.189	9.86	−16.22
DP023211–2	0.9	20408.8	20	2.3	0.64	11161	1848.7	73.67	0.86	17.64	−4.44
	3.0	20446.8	59	6.72	1.06	10747	1726.2	13.2	2.902	17.62	−3.25
	5.0	20448.5	98	11.12	0.94	10787	1736.2	55.9	4.775	6.80	−4.50
DP098140–6	0.9	20448.3	57	6.46	0.61	10840	1748.38	24.89	0.676	12.29	−24.84
	3.0	20445.8	239	27.16	1.5	10836	1747.83	22.78	2.845	5.13	−5.18
	5.0	20436.5	391	44.66	2.53	10818	1744.6	21.56	4.686	5.39	−6.28
DP202216–6	0.9	20460.5	82	9.27	1.17	11838	2000.53	42.46	0.741	16.97	−17.63
	3.0	20460	334	38.13	1.46	11798	1989.87	40.63	3.066	6.52	2.19
	5.0	20414	555	63.84	2.32	11746	1983.21	37.05	5.150	2.91	3.00
MON87403–1	0.9	20450.3	37	4.19	0.46	6431	873.95	8.61	0.959	12.60	6.54
	3.0	19365.3	139	17.41	7.46	6674	869.29	24.76	2.944	6.57	6.57
	5.0	20453.5	213	24.26	1.08	6539	891.76	13.17	4.665	4.74	4.73
MON87411–9	0.9	20409.8	39	4.46	0.89	6275	850.35	17.45	0.877	23.83	−2.58
	3.0	20417.5	141	16.07	1.54	6220	841.09	23.58	3.194	10.62	6.46
	5.0	20436.8	213	24.19	1.27	6250	845	12.96	4.785	5.64	−4.29
MON87429–9	0.9	20436.8	26	2.95	0.33	5876	784.78	18.58	0.733	15.15	−18.59
	3.0	20432.5	94	10.64	2.59	5916	791.34	3.79	2.9444	21.80	−3.93
	5.0	20444.3	181	20.53	1.75	5934	793.71	13.92	4.665	8.13	0.84
MON87460–4	0.9	20445.3	30	3.4	0.75	6424	873.13	20.21	0.913	19.32	−9.28
	3.0	20427.8	93	10.53	0.73	6482	883.74	23.18	2.793	0.73	−20.7
	5.0	20436.3	157	17.82	0.92	6363	863.59	15.88	4.837	0.51	−16.3
MON88017–3	0.9	20450	25	2.83	0.94	6049	762.58	24.01	0.669	30.38	−16.41
	3.0	20420.5	100	11.39	0.24	5946	685.63	94.34	2.744	5.45	−8.5
	5.0	20431.5	166	18.91	0.39	5956	757.17	24.03	4.551	3.62	−9.0

^a^Each value represents the average of four experimental replications, except for SD, precision, and trueness. ^b^ GM % in copy/maize haploid genome copy number × 100. SD, standard deviation of Conc. Cp/µl (concentration of copy/µl); RSD, relative standard deviation; Bias, deviation from expected value.

Additionally, GM content was calculated using a zygosity ratio of 0.5, assuming that maize is in the market predominantly as a hybrid trait (Supplemental Table S5). For some GMO contents of DAS01131–3, DP04114–3, DP023211–2, DP051291–1, DP202216–6, MON87411–9, and MON88017–3, the % RSD and % bias did not meet the acceptance criteria, indicating that accurate determination of the zygosity ratio is crucial for precise GMO quantification.

In conclusion, duplex cdPCR demonstrated comparable performance to qPCR in quantifying DP023211–3. Determination of the zygosity ratio of GM DNA samples was crucial for accurate quantification of GM content. The duplex cdPCR method achieved accurate quantification of twelve GM maize events at GMO levels as low as 0.9% and successfully quantified 3.0% and 5.0% GM content with good precision and trueness. These findings demonstrate the duplex cdPCR method is straightforward and does not require additional optimization trials, unlike qPCR, which requires standard curve establishment. While the present study focused on key performance metrics such as accuracy and precision, we acknowledge that a comprehensive in-house validation will be necessary to support broader regulatory application. Future work will therefore include a full validation of the method in accordance with ENGL and other international guidelines, addressing essential parameters such as limit of detection (LOD), LOQ, robustness, and reproducibility. We also plan to explore participation in inter-laboratory ring trials to further assess the method’s reliability and transferability.

## Supplementary Material

Kim et al_Supplemental Information_clean.docx
