# Supplementary material for: Development of a duplex chamber digital PCR to quantify twelve genetically modified maize events
Source: GM Crops Food. 2025 Aug 21;16(1):527–38. doi: 10.1080/21645698.2025.2548053 (PMC12377078; doi:10.1080/21645698.2025.2548053)
Supplement: Kim et al_Supplemental Information_clean.docx [file KGMC_A_2548053_SM6672.docx]

Supplemental Table 1. Information on fourteen GM maize events used in this study, including approval year in South Korea, intended use, trait description, and developer

| Event | Approval year | | | Commercial trait | GM trait | Developer |
| --- | --- | --- | --- | --- | --- | --- |
|  | Food | Feed | Industrial use |  |  |  |
| DAS01131-3 | 2024 | 2024 |  | Herbicide Tolerance,  Insect Resistance | Glyphosate herbicide tolerance,  Lepidopteran insect resistance | Corteva Agriscience |
| DAS40278-9 | 2014 | 2014 | 2021 | Herbicide Tolerance | Herbicide Tolerance | [Dow AgroSciences LLC](https://www.isaaa.org/gmapprovaldatabase/developedby/default.asp?DeveloperID=13&DevelopedBy=Dow%20AgroSciences%20LLC) |
| DAS59122-7 | 2006 | 2008 | 2021 | Herbicide Tolerance,  Insect Resistance | Glufosinate herbicide tolerance,  Coleopteran insect resistance | [Dow AgroSciences LLC and DuPont (Pioneer Hi-Bred International Inc.)](https://www.isaaa.org/gmapprovaldatabase/developedby/default.asp?DeveloperID=25&DevelopedBy=Dow%20AgroSciences%20LLC%20and%20DuPont%20(Pioneer%20Hi-Bred%20International%20Inc.)) |
| DP004114-3 | 2005 | 2005 | 2021 | Herbicide Tolerance,  Insect Resistance | Glufosinate herbicide tolerance, Coleopteran insect resistance, Lepidopteran insect resistance | DuPont (Pioneer Hi-Bred International Inc.) |
| DP023211-2 | 2024 | 2024 |  | Herbicide Tolerance,  Insect Resistance | Glufosinate herbicide tolerance, Coleopteran insect resistance , Mannose metabolism | Dow AgroSciences LLC and DuPont (Pioneer Hi-Bred International Inc.) |
| DP098140-6 | 2010 | 2010 | 2021 | Herbicide Tolerance | Glyphosate herbicide tolerance,  Sulfonylurea herbicide tolerance | DuPont (Pioneer Hi-Bred International Inc.) |
| DP202216-6 | 2023 | 2022 |  | Herbicide Tolerance,  Altered Growth/Yield | Glufosinate herbicide tolerance,  Enhanced Photosynthesis/Yield | [Dow AgroSciences LLC](https://www.isaaa.org/gmapprovaldatabase/developedby/default.asp?DeveloperID=13&DevelopedBy=Dow%20AgroSciences%20LLC) |
| MON87403-1 | 2017 | 2016 | 2021 | Altered Growth/Yield | Altered Growth/Yield | [Monsanto Company (including fully and partly owned companies)](https://www.isaaa.org/gmapprovaldatabase/developedby/default.asp?DeveloperID=22&DevelopedBy=Monsanto%20Company%20(including%20fully%20and%20partly%20owned%20companies)) |
| MON87411-9 | 2016 | 2016 | 2021 | Herbicide Tolerance,  Insect Resistance | Glyphosate herbicide tolerance,  Coleopteran insect resistance | [Monsanto Company (including fully and partly owned companies)](https://www.isaaa.org/gmapprovaldatabase/developedby/default.asp?DeveloperID=22&DevelopedBy=Monsanto%20Company%20(including%20fully%20and%20partly%20owned%20companies)) |
| MON87429-9 | 2023 | 2023 |  | Herbicide Tolerance | Glufosinate herbicide tolerance , Glyphosate herbicide tolerance , Dicamba herbicide tolerance , 2,4-D herbicide tolerance | Bayer Australia |
| MON87460-4 | 2012 | 2011 | 2021 | Abiotic Stress Tolerance | Drought stress tolerance , Antibiotic resistance | Monsanto Company and BASF |
| MON88017-3 | 2006 | 2006 | 2021 | Herbicide Tolerance,  Insect Resistance | Glyphosate herbicide tolerance,  Coleopteran insect resistance | [Monsanto Company (including fully and partly owned companies)](https://www.isaaa.org/gmapprovaldatabase/developedby/default.asp?DeveloperID=22&DevelopedBy=Monsanto%20Company%20(including%20fully%20and%20partly%20owned%20companies)) |

Supplemental Table 2. qPCR reaction components and final concentrations used for quantification of DP023211-2 and *hmg*

| Component | Final concentration | µL/reaction |
| --- | --- | --- |
| Qplex Master Mix (2x) | 1x | 12.5 |
| Forward primer (10 µM) | 400 nM | 1 |
| Reverse primer (10 µM) | 400 nM | 1 |
| Probe (10 µM) | 100 nM | 0.25 |
| Nuclease free water | - | 1.25 |
| DNA | - | 10 |
| Total reaction volume |  | 25 µL |

Supplemental Table 3. Copy number values for standard curve samples prepared for DP023211-2 quantification by qPCR

| Sample code | S1 | S2 | S3 | S4 | S5 |
| --- | --- | --- | --- | --- | --- |
| Total amount of maize DNA in reaction (ng) | 300 | 75 | 25 | 6.25 | 1.56 |
| DP023211-2 copies | 109,89 | 2,747 | 916 | 229 | 57 |
| Haploid genome copies | 109,890 | 27,473 | 9,158 | 2,289 | 572 |

Supplemental Table 4. Comparison of zygosity ratios measured by duplex cdPCR and reported by applicants or EURL-GMFF

| Event | This study | Application report | EURL-GMFF |
| --- | --- | --- | --- |
| DAS01131-3 | 0.399 | 0.579 | 0.59 |
| DAS40278-9 | 0.408 | ‒ | 0.36 |
| DAS59122-7 | 0.424 | ‒ | ‒ |
| DP004114-3 | 0.356 | ‒ | 0.58 |
| DP023211-2 | 0.134 | 0.42 | ‒ |
| DP098140-6 | 0.546 | ‒ | ‒ |
| DP202216-6 | 0.625 | 0.58 | 0.99 |
| MON87403-1 | 0.583 | ‒ | 0.58 |
| MON87411-9 | 0.598 | ‒ | 0.62 |
| MON87429-9 | 0.513 | ‒ | 0.538 |
| MON87460-4 | 0.427 | ‒ | ‒ |
| MON88017-3 | 0.521 | ‒ | ‒ |

‒ Zygosity ratio value is not available in the validation report by either the applicant or European Union Reference Laboratory for Genetically Modified Food and Feed (EURL GMFF).

Supplemental Table 5. Measured GMO content using a fixed zygosity ratio of 0.5 for twelve GM maize events^a^

| Event |  | Total | Event specific target | | |  | *hmg* | | |  |  |  |
| --- | --- | --- | --- | --- | --- | --- | --- | --- | --- | --- | --- | --- |
|  | ^b^Target  GM-levels % |  | Positive | Conc.cp/µl | SD |  | Positive | Conc.cp/µl | SD | ^a^Measured GM-level % | Precision (RSD%) | Trueness (bias %) |
| **DAS01131-3** | 0.9 | 20426.3 | 26 | 2.98 | 0.57 |  | 7373 | 1037.1 | 47.96 | **0.57** | **27.89** | **-36.14** |
|  | 3.0 | 20455.5 | 111 | 12.54 | 0.61 |  | 7563 | 1068.7 | 24.23 | 2.35 | 6.19 | -21.77 |
|  | 5.0 | 20445.3 | 191 | 21.75 | 1.49 |  | 7742 | 1101.8 | 23.17 | 3.95 | 9.51 | -21.03 |
| DAS40278-9 | 0.9 | 20463.3 | 59 | 6.71 | 0.86 |  | 10382 | 1638.8 | 19.29 | 0.82 | 15.69 | -9.01 |
|  | 3.0 | 20474.8 | 190 | 21.58 | 1.22 |  | 10296 | 1618.0 | 31.34 | 2.67 | 4.54 | -11.08 |
|  | 5.0 | 20453.5 | 294 | 33.54 | 0.78 |  | 10237 | 1606.9 | 27.63 | 4.17 | 3.84 | -16.51 |
| DAS59122-7 | 0.9 | 20361 | 46 | 6.46 | 0.61 |  | 9208 | 1393.4 | 27.13 | 0.74 | 11.60 | -17.39 |
|  | 3.0 | 20437.5 | 157 | 27.16 | 1.5 |  | 9147 | 1373.7 | 26.96 | 2.59 | 3.61 | -13.52 |
|  | 5.0 | 20417.8 | 288 | 44.64 | 2.53 |  | 9165 | 1379.2 | 18.29 | 4.76 | 2.87 | -4.78 |
|  | 0.9 | 20457 | 48 | 5.44 | 0.36 |  | 12447 | 2170.9 | 47.2 | **0.50** | **6.59** | **-44.31** |
| **DP004114-3** | 3.0 | 20450.5 | 176 | 19.98 | 1.08 |  | 12423 | 2164.9 | 41.5 | **1.85** | **49.34** | **-38.47** |
|  | 5.0 | 20462.5 | 285 | 32.44 | 2.46 |  | 12468 | 2176.2 | 54.4 | **2.98** | **9.86** | **-40.37** |
| **DP023211-2** | 0.9 | 20408.8 | 20 | 2.3 | 0.64 |  | 11161 | 1848.7 | 277.9 | **0.25** | **17.01** | **-72.35** |
|  | 3.0 | 20446.8 | 59 | 6.72 | 1.06 |  | 10747 | 1726.2 | 13.2 | **0.78** | **17.62** | **-74.05** |
|  | 5.0 | 20448.5 | 98 | 11.12 | 0.94 |  | 10787 | 1736.2 | 55.9 | **1.28** | **6.80** | **-74.38** |
| DP098140-6 | 0.9 | 20448.3 | 57 | 6.46 | 0.61 |  | 10840 | 1748.38 | 24.89 | 0.74 | 12.29 | -17.89 |
|  | 3.0 | 20445.8 | 239 | 27.16 | 1.5 |  | 10836 | 1747.83 | 22.78 | 3.11 | 5.86 | 3.60 |
|  | 5.0 | 20436.5 | 391 | 44.66 | 2.53 |  | 10818 | 1744.6 | 21.56 | 5.12 | 5.39 | 2.40 |
| **DP202216-6** | 0.9 | 20460.5 | 82 | 9.27 | 1.17 |  | 11838 | 2000.53 | 42.46 | 0.93 | 16.97 | 2.97 |
|  | 3.0 | 20460 | 334 | 38.13 | 1.46 |  | 11798 | 1989.87 | 40.63 | **3.83** | **6.30** | **27.75** |
|  | 5.0 | 20414 | 555 | 63.84 | 2.32 |  | 11746 | 1983.21 | 37.05 | **6.44** | **2.91** | **28.76** |
| MON87403-1 | 0.9 | 20450.3 | 37 | 4.19 | 0.46 |  | 6431 | 873.95 | 8.61 | 0.96 | 12.60 | 6.54 |
|  | 3.0 | 19365.3 | 139 | 17.41 | 7.46 |  | 6674 | 1014.04 | 251.3 | 3.43 | 17.54 | 14.46 |
|  | 5.0 | 20453.5 | 213 | 24.26 | 1.08 |  | 6539 | 891.76 | 13.17 | 5.44 | 4.74 | 8.82 |
| **MON87411-9** | 0.9 | 20409.8 | 39 | 4.46 | 0.89 |  | 6275 | 850.35 | 17.45 | 1.05 | 23.83 | 16.55 |
|  | 3.0 | 20417.5 | 141 | 16.07 | 1.54 |  | 6220 | 841.09 | 23.58 | **3.82** | **11.80** | **27.37** |
|  | 5.0 | 20436.8 | 213 | 24.19 | 1.27 |  | 6250 | 845 | 12.96 | 5.73 | 6.08 | 14.51 |
| MON87429-9 | 0.9 | 20436.8 | 26 | 2.95 | 0.33 |  | 5876 | 784.78 | 18.58 | 0.75 | 15.15 | -16.47 |
|  | 3.0 | 20432.5 | 94 | 10.64 | 2.59 |  | 5916 | 791.34 | 3.79 | 2.96 | 21.80 | -1.43 |
|  | 5.0 | 20444.3 | 181 | 20.53 | 1.75 |  | 5934 | 793.71 | 13.92 | 5.17 | 8.13 | 3.46 |
| MON87460-4 | 0.9 | 20445.3 | 30 | 3.4 | 0.75 |  | 6424 | 873.13 | 20.21 | 0.69 | 19.32 | -23.47 |
|  | 3.0 | 20427.8 | 93 | 10.53 | 0.73 |  | 6482 | 883.74 | 23.18 | 2.38 | 0.73 | -20.56 |
|  | 5.0 | 20436.3 | 157 | 17.82 | 0.92 |  | 6363 | 863.59 | 15.88 | 4.13 | 0.51 | -17.46 |
| **MON88017-3** | 0.9 | 20450 | 25 | 2.83 | 0.94 |  | 6049 | 762.58 | 24.01 | 0.78 | **30.38** | -12.91 |
|  | 3.0 | 20420.5 | 100 | 11.39 | 0.24 |  | 5946 | 685.63 | 94.34 | 2.86 | 5.45 | -4.7 |
|  | 5.0 | 20431.5 | 166 | 18.91 | 0.39 |  | 5956 | 757.17 | 24.03 | 4.74 | 3.62 | -5.2 |

^a^ Each value represents the average of four experimental replications, except for SD, precision, and trueness. ^b^ GM % in copy/maize haploid genome copy number × 100. SD, standard deviation of Conc. Cp/µl (concentration of copy/µl); RSD, relative standard deviation; Bias, deviation from expected value. Bold text indicates GM-level % values that did not meet the acceptance criteria of precision **(±25% RSD)** and trueness **(≤25% bias), when a fixed zygosity ratio of 0.5 was used.**


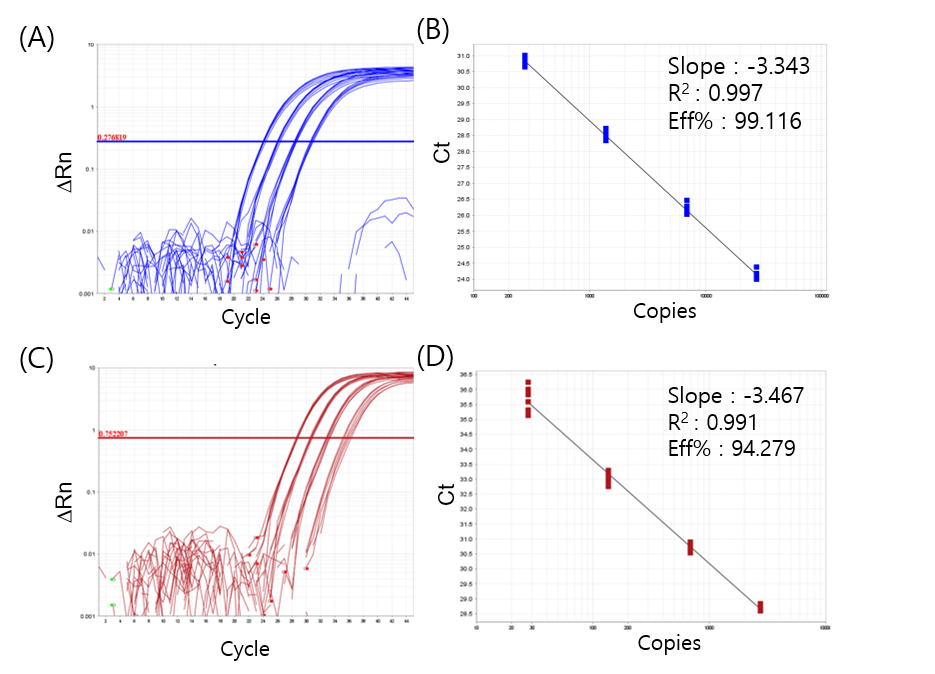


Supplemental Figure 1. Amplification plots and standard curves of DP023211-2 by qPCR analysis. Zygosity ratio 0.43 was applied. (A) Amplification curves for endogenous *hmg* gene. (B) The standard curve of DP023211-2 with concentrations from 38 to 7326 copies. (C) qPCR amplification curves for specific target sequence of DP023211-2. (D) The standard curve of *hmg* with concentrations from 380 to 73260 copies. Four replicates were performed at each concentration.
